# Unveiling dynamic interactions: in vivo imaging chronicles inflammation and regeneration in living organisms

**DOI:** 10.1186/s41232-023-00312-3

**Published:** 2023-12-12

**Authors:** Kenji Kabashima

**Affiliations:** 1 Department of Dermatology, Kyoto University Graduate School of Medicine, 54 Shogoin Kawahara-cho, Sakyo, Kyoto, 606-8507 Japan; 2 A*STAR Skin Research Labs (A*SRL) and Skin Research Institute of Singapore (SRIS), Agency for Science, Technology and Research (A*STAR), 8A Biomedical Grove, #06-06 Immunos, Singapore, Republic of Singapore

To the Editor,

I am pleased to present a Thematic Series “In vivo imaging dissecting inflammation and regeneration of living body” that delves into the intricacies of inflammation and regeneration through the lens of cutting-edge in vivo imaging techniques. The three papers compiled in this series offer a comprehensive exploration of inflammatory responses within various physiological contexts, shedding light on the dynamic interplay between cells in living organisms.

## In vivo imaging of inflammatory response in cancer research

The first paper, authored by Konishi Y and Terai K, provides a detailed examination of the role of inflammation in the development and progression of cancer [1]. Recognizing the complex nature of intercellular cross-talk within the tumor microenvironment, the authors leverage two-photon excitation microscopy (2P-IVM) to unravel the spatiotemporal dynamics of these dynamic events. Through the use of fluorescent indicators of signal transduction, Konishi and Terai present a thorough review of current 2P-IVM techniques, emphasizing its role in elucidating cross-talks between cancer cells and surrounding immune and non-immune cells. The integration of red-shifted indicators and optogenetic tools into 2P-IVM is also discussed, highlighting the technique’s ongoing relevance in providing the much-needed spatiotemporal context in the field of cancer research.

## Intravital imaging of immune responses in intestinal inflammation

The second paper, authored by Honda M et al. focuses on the critical role of intravital microscopy in unraveling the mysteries of immune responses within the gastrointestinal tract [2]. The authors emphasize the necessity of understanding the in vivo behavior of immune cells in various gastrointestinal conditions, including inflammation, colitis, inflammatory bowel disease, ischemia–reperfusion injury, and neutrophil extracellular traps. Through the lens of intravital microscopy, Honda et al. explore the emerging role of this technique in providing novel insights into intestine-specific events, shedding light on both innate and adaptive immunities. The review not only outlines the application of intravital imaging in gastrointestinal research but also highlights the novel findings obtained through this innovative approach.

## Localization and movement of Tregs in gastrointestinal tract: a systematic review

The third paper, a systematic review authored by Harada Y et al. addresses the localization and movement of regulatory T cells (Tregs) in the gastrointestinal tract [3]. Acknowledging the essential role of Tregs in preventing systemic autoimmune diseases and inhibiting inflammation, the authors utilize in vivo imaging technology to visualize how Tregs localize and move in settings of inflammation and homeostasis. The review underscores the significance of Treg characterization based on their location, revealing that Tregs in different parts of the gastrointestinal tract induce tolerance against specific antigens, influencing immune homeostasis. The paper not only summarizes the induction of Tregs in the digestive tract but also outlines the application of in vivo Treg imaging in elucidating immune homeostasis.

In conclusion, this Thematic Series offers a panoramic view of the in vivo imaging landscape, showcasing its pivotal role in dissecting inflammation and regeneration within living organisms. These papers contribute not only to the current body of knowledge but also pave the way for future advancements in our understanding of dynamic cellular interactions in health and disease.
